# Acceptability and utility of parental guidance on weight talk with children for GPs: a qualitative study

**DOI:** 10.3399/BJGPO.2025.0087

**Published:** 2026-06-03

**Authors:** Billy Twomey, Fiona Gillison

**Affiliations:** 1 Irish College of General Practitioners, Dublin, Republic of Ireland; 2 Department for Health, University of Bath, Bath, UK

**Keywords:** qualitative research, childhood obesity, general practice

## Abstract

**Background:**

Childhood overweight and obesity rates are rising globally, yet many healthcare professionals and parents worry that raising the issue could harm children’s wellbeing. New, evidence-informed guidance for parents on constructive ways to talk to children about their weight has been created to address these concerns. GPs are important in providing and endorsing this guidance, as part of broader efforts to support families.

**Aim:**

To explore the acceptability and usability of this novel guidance from the perspective of general practice.

**Design & setting:**

A qualitative study with GPs in the Republic of Ireland.

**Method:**

Semi-structured interviews with practising GPs were audio recorded, transcribed verbatim, and thematically analysed.

**Results:**

Fifteen GPs (10 male, five female) participated. Three key themes were identified. Theme 1 explored how the guidance was deemed to be useful in overcoming perceived barriers to having conversations with parents about their child’s weight. Participants believed it would be effective in reducing stigma, promoting conversations and providing a framework to the consultation. Theme 2 explored how the guidance could be integrated into practice, supporting an individualised and patient-centred approach. Theme 3 explored the need for a multidisciplinary approach to the complex area of supporting children with overweight and obesity, and the need to focus on consistent evidence-based information.

**Conclusion:**

The guidance was perceived as acceptable, relevant, and useful. GPs felt that evidence-based resources, like this, are needed to provide consistent and accurate information for children and parents with regards to the management of overweight and obesity.

## How this fits in

Childhood overweight and obesity remains a significant public health concern, yet GPs often report challenges in initiating weight-related discussions with parents. Lack of evidence-based support to provide parents can contribute to concerns about raising the topic. A qualitative study was conducted with GPs to explore the acceptability and usability of a new evidence-informed guidance on talking to children about weight. The guidance was perceived to facilitate effective communication in challenging consultations by reducing stigma, providing a structured framework, and incorporating input from key stakeholders.

## Introduction

Obesity is a complex, multifactorial condition affecting children of all age groups.^1^ With prevalence rates increasing worldwide, a major global health challenge exists.^2^ High rates of concern are attributable to the negative physical, psychological, and social sequelae of excess weight in childhood.^3^ As GPs are often the first point of contact for children and their families, childhood obesity represents a significant issue in primary care.^4^ GPs’ provision of ongoing, continuous care is particularly important, given that most children living with obesity continue to do so into adulthood.^5^

Healthcare professionals (HCPs), including GPs, have an important role to play in both the prevention and management of childhood obesity. However, the opportunity for clinical intervention remains underutilised.^6^ Many HCPs feel incompetent at discussing childhood obesity in consultations.^7,8^ Barriers include lack of training, concerns of triggering eating disorders, and anxiety of potential conflict with families.^9^ In addition, clinicians are less likely to raise sensitive topics such as weight when they do not have practical support or resources to offer. The availability of actionable, evidence-based interventions has been shown to increase the likelihood of initiating such discussions.^10^

While some parents respond negatively to initial feedback from a clinician that their child is overweight, they typically recognise a legitimate role for GPs in supporting them with children’s weight management.^11^ Qualitative research has shown that parents often feel responsible for their child’s weight and acknowledge that regular weight check-ups can be important.^12^ Furthermore, parents have reported feeling isolated in handling their child’s weight, especially when their concerns were not addressed by HCPs.^13^ These findings suggest a role for the collaborative involvement of both parents and HCPs, particularly GPs, in discussions with children surrounding their weight. They also point to the need for greater confidence among GPs in managing consultations surrounding childhood obesity.

Until recently, evidence-based guidance for both HCPs and parents on how to talk to children about weight has been limited. In 2022, new guidance was co-created in the UK^14^ and recommended for sharing with parents as part of the National Child Measurement Programme (NCMP).^15^ Developed primarily for parents and caregivers, the guidance was created in response to concerns that weight-related conversations could harm children’s self-esteem and wellbeing,^16^ contributing to low parental engagement in weight management services.^17^ Although the guidance is designed for parents, HCPs share parents’ concerns about the risks of talking to children about weight.^18^ While some forms of weight-talk between parents and children are associated with poor wellbeing, this is not always the case.^19^ We propose that HCPs may feel reassured and more confident in raising the topic if supported by evidence-informed guidance that supports parents to engage in constructive conversations with their children.

Feedback from parents and school nurse teams has been positive, suggesting that the guidance is both acceptable and useful, and may have value for all HCPs working with families and children living with obesity.^18^ However, its acceptability and application in general practice have not yet been explored. The aim of this study was to explore GPs’ views on whether, and how, the guidance could support their practice when working with parents and children with overweight or obesity.

## Method

### Design

This was a qualitative interview study conducted from August 2023 to March 2024.

### Participants and recruitment

Actively practising GPs with a minimum of 12 months clinical experience were eligible for the study. Recruitment was achieved via internal mail among the Irish College of General Practitioners Trinity College Dublin (TCD) training network. This broad network includes GPs practising across urban, rural, affluent, and more deprived areas within the province of Leinster, providing a diverse and sufficient participant pool without the need to expand beyond the network. GPs were emailed an overview of the study and its objectives, and details on how to express interest in taking part. Volunteers were emailed a link to the full participant information sheet and consent forms and given the opportunity for questions. Once consent was obtained, participants were asked to read and review the guidance prior to interview and were given a minimum of 1 week to do this.

### Data collection

Participants were interviewed via video call by BT between August 2023 and March 2024. A broad semi-structured interview topic guide was informed by literature and our experience of talking to HCPs in prior work.^18^ The interview topic guide included open and broad questions to allow flexibility (see supplementary file), and was piloted with one GP to evaluate suitability of the questions before it was finalised; no amendments were needed. The interview schedule was divided into two parts. The first explored GPs’ attitudes and beliefs surrounding consultations with or about children with overweight or obesity. The second part focused on the guidance document itself with exploration of both positive and negative feedback, and an ultimate goal of assessing acceptability and usability of this guidance in primary care.

### The guidance

The guidance entitled *Talking to your child about weight – A guide for parents and caregivers of children aged 4-11 years* was developed through a systematic, collaborative process combining evidence review, empirical research and a Delphi-style study including parents, children, HCPs (including GPs), academics, and public health specialists.^14^ It is available in the supplementary file. The resource is designed to support conversations between parents and their children about their weight and promote healthy behaviours while avoiding negative effects like stigma or body dissatisfaction. The guidance has been promoted to GPs (and other HCPs) as a tool they can provide to parents to support their work with families, and has been endorsed through publication by the British Dietetic Association (BDA) and dissemination from public health bodies including The Office for Health Improvement and Disparities (OHID) in England. The guide consists of seven distinct sections addressing different questions parents may have and providing information and suggestions for parents on how to talk to their children about weight. These sections are namely:

1.  Should I talk to my child about weight?
2.  The whole family counts
3.  Top tips for talking to your child about weight
4.  How to help your child feel good about their body
5.  What if I am struggling with my own weight?
6.  What could I say when …?
7.  Links to further advice and information

The text within the guidance is displayed in bullet points and broken up by quotes and positive images of children and families. Narrative messages and case studies are included to improve accessibility of the content for those with lower educational levels, and reduce negative reactance (such as, defensiveness against unwelcome messages).^20^ The participants of this study were sent the guidance in PDF format via email, following receipt of the consent forms.

### Data analysis

All interviews were audio recorded and transcribed verbatim for analysis. Any information that could identify participants, patients, or families was omitted as outlined on the participant information leaflet. Participants were allocated unique identification numbers and no identifiable data were stored. Audio recordings were password protected and deleted once corresponding transcripts had been uploaded to the secure server at the University of Bath. The research team engaged in reflective practice throughout analysis to recognise and address potential biases.

All anonymised interview transcripts were imported to NVivo (version 14). A thematic analysis was conducted to allow identification, analysis, and reporting of repeated patterns. Thematic analysis is seen as an appropriate and powerful method to use when seeking to understand a set of experiences, thoughts, or behaviours across a data set.^21^ This was pertinent to our research objective. The transcripts were read multiple times by BT to obtain data familiarisation. Initial codes were then generated from the text to explore relevant and interesting data points. After the first two transcripts had been coded, a discussion between BT and FG took place to refine and review the coding scheme. This coding schedule was subsequently applied to the remaining transcripts with a flexible and iterative approach to ensure codes consistently reflected the data. All transcripts were coded individually, and a combined inductive and deductive approach was used to identify patterns that were salient to the research objectives. Clusters of codes on similar findings and patterns were organised into potential themes. These themes were discussed among BT and FG, continuously compared with the original data, and revised and refined to accurately capture the priorities of the research.

Fifteen interviews were conducted as part of the study and 10 male and five female GPs participated. Clinical experience ranged from 18 months to 30 years of working in general practice (mean = 9.2 years, median = 6 years). Interview duration ranged from 27 to 58 minutes in length (mean = 39, median = 38). All 15 interviews were conducted by videocall. Where interview extracts are shown the participant has been assigned a numerical identifier, for example, GP1.

## Results

The guidance document was unanimously well received by the participating GPs. All 15 participants found it to be relevant to clinical practice and agreed that providing care to children with overweight and obesity is a significant and growing challenge. Responses were summarised into the following three themes.

### Theme 1: Perceived usability of the guidance document in primary care

#### Removing barriers to conversations about weight

Participants noted a number of barriers to having consultations with parents about children’s weight and obesity. Most GPs reported time constraints as a significant barrier, finding busy clinics and short appointment times as obstructive to discussing weight with children and parents. The majority noted that children are rarely booked in specifically for discussions about weight, with consultations more commonly focused on acute concerns such as coughs or skin rashes. As a result, there is often limited time to address weight-related issues, despite GPs recognising their importance. Many expressed a willingness to raise the topic opportunistically but find that time limitations frequently prevent them from doing so:

*’Time pressure is such a challenge in general practice. You have to primarily address the reason the parents have brought their child in for and if you then bring up weight as a separate issue you could be really stuck* …’ (GP4)

Another commonly described barrier was that of a paucity of resources and training in this area. Most GPs that participated could not name a resource they use when they do have these consultations:

*’The advice I give them doesn’t extend much further than the very basic eat well, exercise more, and monitor sleep. We need better resources available …*’ (GP7)

The GPs interviewed unanimously felt that the evidence-informed guidance provided within this study was useful in overcoming these barriers, among others. They described potential for it to enhance their knowledge and confidence, reduce fear of conflict in the doctor–patient relationship, and improve their awareness of appropriate and effective communication strategies. Furthermore, some GPs felt the guidance could improve efficiency by providing structure to conversations about weight:

*’It helps with awkwardness because you have something to support what you are saying and that tells you that it is okay to talk about this. It sends positive messages to the parents so that they don’t get angry at you for bringing this up. It can also be used as a tool to provide structure in the consultation which in turn can save time*.’ (GP15)

### Promoting conversations

Many GPs reported that they actively avoid asking about weight in children due to fear of confrontation from the parents and negatively affecting the doctor–patient relationship. The perceived stigma surrounding overweight and obesity provoked anxiety in many GPs, particularly when parents are not presenting to discuss this area. Even those who reported that they tend not to broach the topic, also acknowledged that this is their role and that promoting a healthy childhood weight is a significant issue facing primary care:

*‘Being honest, I usually ignore it. I don’t think I’ve ever fully broached it. I have been close and skirted around it a few times but I just have this fear of how the parent would take it and also fear of doing it in front of the child.*’ (GP13)

GPs with more experience and long-term relationships with patients often had lower levels of anxiety than those who were earlier in their careers or had not met the families before. However, all participating GPs felt that the guidance document helped overcome potential anxiety and awkwardness in having these conversations. Additionally, a number of participants suggested that dissemination of the guidance might prompt parents to book weight-specific consultations with their children:

*’It helps to reduce stigma and opens up the whole area for discussion. If we can get more people talking openly about weight, then that’s a huge step. It doesn’t directly tell parents to talk to their kids about weight, but it gives ways that can be supportive and how to respond when opportunities might come up.*’ (GP10)

## Theme 2: Integration into clinical practice

### Format and content

Participants suggested a variety of different formats in which the guidance may be used in practice. The most common preference was for a printed version that could be handed to the patients, but there was also support for online web pages, uploaded PDFs to the practice website, mobile applications, and interactive e-learning modules.

GPs also appreciated the document’s content and accessible language. They found the tone encouraging, fostering discussions. Visual elements, font, and case studies were well received, ensuring suitability for both parents and children. A whole-family approach emerged as a core value, aligning with GP perspectives:

*‘It comes down to what way you think the parents and families are going to take it on board. Traditionally paper print-outs were used but some prefer direct links sent to their phone nowadays. You want it to be accessible to the user.*’ (GP9)

*‘The language would generally be considered accessible and user friendly. However, we have a few patients with literacy issues, and I would have to read this to them or else look at an audio option*.’ (GP3)

One-third of GPs interviewed mentioned that a document 20 pages long may be initially daunting to the reader, even if they agreed that the content was made accessible through the use of sections, pictures, and bullet points:

*‘The length initially looked a bit long but once you started to read through it, you quickly realised it was reasonable. The sections broke it up nicely and meant you could leave it and come back at different points*.’ (GP6)

### Methods of use

Numerous GPs proposed using the guidance document as an ‘icebreaker’ to help begin conversations about the child’s weight. Others suggested giving it to the parent at the end of the consultation to support key messages conveyed by the GP. Many participants also saw it as a way to encourage parents to arrange dedicated appointments to talk about a child’s weight:

*‘Having a tool like this would certainly help me as I could give them this to look at and they could come back to me. This would help reduce awkwardness and show that I am not having an agenda.’* (GP14)

One participant suggested that GPs could use the guidance to provide education sessions to the patients of the practice, as well as part of their continuing professional development (CPD) group. At a dedicated tutorial session, the guidance document would be provided to interested parents and/or GPs beforehand and either an in-person or online education session would be offered. Other suggestions included the use of QR codes that would direct users to the guidance document, enhancing its dissemination.

## Theme 3: Role of the GP

### Multidisciplinary approach

Participants unanimously felt that the GP has a significant role in the management of children with overweight and obesity. Assessment and diagnosis, education and counselling, formulation of management plans, and patient advocacy were all mentioned as roles of the GP. Less experienced GPs demonstrated a greater tendency to prioritise investigating underlying causes before considering therapeutic options, whereas more experienced GPs were more inclined to integrate investigations into a comprehensive management plan from the same consultation. Participating GPs also noted that other healthcare professionals are equally important (including practice nurses, dieticians, public health nurses and physicians, physiotherapists, psychologists, social workers, speech and language therapists, and occupational therapists), and that the guidance document should be available to all those involved and used in health settings beyond primary care:

*‘The GP 100% has a role. We should be looking out for this and screening for complications. However, I do think there is a significant role for other members of the team too. Our practice nurse has longer appointments than me and is excellent at providing education to patients. I would certainly want her to have this guidance’.* (GP5)

*‘I also think this would work really well in the hospitals and out in the community, such as in schools. The more people it reaches, the better. The more readers, the more awareness and more buy-in for an evidence-based approach.’* (GP2)

The need for consistent and evidence-based information was a recurring message throughout the interviews. Participants frequently reported concerns about sources of misinformation, most notably through social media accounts and unregulated online practitioners. Participants who referenced sources of misinformation, unanimously felt the guidance document helped to overcome this:

*‘It’s really important that children and adults know there is so much misinformation out there. Social media is the most topical source at the moment … I liked how the guidance mentioned this and that kids are going to hear about weight from other sources. As healthcare practitioners we should be providing clarity on this area. We need to educate the kids, and this guidance tells us that they will be receptive to that.’* (GP15.)

Despite the universal acceptance of a multidisciplinary approach, many of the participants discussed frustrations at delays and waiting times for referrals to other healthcare members. They suggested that section 7 of the guidance document, which provided links to further information and resources, was particularly useful as avenues to signpost families while awaiting further review:

*‘Making this guidance document available and accessible can help for those who are waiting. The links to further information and resources section was really useful*.‘ (GP12)

## Discussion

### Summary

This study provides novel insights into the perceptions of practising GPs in the Republic of Ireland regarding the acceptability and usability of a new evidence-informed guidance document for parents and caregivers on talking to children about weight. Three key themes were identified. Theme 1 explored GPs’ beliefs that the guidance could address an important barrier GPs identified to conversations about children’s weight, caused by the lack of training and resources. Interviewees felt the guidance enhanced their knowledge and confidence and that the compassionate way that the information was framed would reduce their concerns about the potential for conflict with parents. In Theme 2, GPs confirmed a preference having the guidance available in hard copy to hand out to patients, potentially ahead of a consultation about a child’s weight to set a positive tone for the discussion. They also believed digital versions suitable for a practice website would be useful, as well as formats that could be used for GP training. In Theme 3, GPs talked about their role in the promotion of children’s healthy weight as part of a wider multidisciplinary team, and the potential for the guidance to provide a trusted source of information for parents in the face of conflicting information on social media.

### Strengths and limitations

The qualitative approach enabled in-depth exploration of diverse perspectives from 15 GPs regarding the guidance. Strengths include recruiting GPs across a range of ages and experience levels, supporting the generalisability of findings on usability.

Limitations include potential selection bias, as participants may have had pre-existing interest in the topic, potentially leading to more positive views. GPs less engaged with childhood weight discussions may hold different perspectives. The study also captures anticipated rather than actual use of the guidance in practice.

The lead researcher, a practising GP, conducted the interviews. This facilitated recruitment, rapport, and context-sensitive interpretation, but shared professional experiences may have reduced challenge to assumptions or biases. The lead author was independent of the guidance creation. To minimise bias from the second author, who led guidance development, participant recruitment, interviews, and coding were conducted independently.

### Comparison with existing literature

The barriers GPs reported to discussing children’s weight were consistent with past work.^22,23^ These included time limitations, fear of conflict with patients, and uncertainty about how to support parents. Prior to the development of the guidance, resources targeting communication between GPs (and primary care professionals) and parents were available; the guidance available from Public Health England at the time (*Let’s talk about weight: A step-by-step guide to conversations about weight management with children and families for health and care professionals*, PHE, 2017) has since been replaced by NICE Guideline 246.^24,25^ However, GPs’ confidence remains low,^26^ indicating a need for further support and training. The positive response from GPs suggests that in being designed specifically to be shared with parents, unlike previous resources, this need is better met. For example, GPs felt that the guidance could be an icebreaker with parents by setting out the compassionate, anti-stigmatising tone they intended for their consultation, and could therefore reduce barriers related to fear of conflict with parents.

While the study was not designed to differentiate responses by GP characteristics, there was some evidence that the guidance may have a greater impact on recently qualified GPs. More experienced participants emphasised the importance of existing trusted relationships with families, whereas less experienced GPs identified fear or conflict as greater barriers. This supports calls for early training for healthcare professionals on communicating with families about weight.^27^

Across themes, participants provided ideas as to how the new guidance could be used operationally within general practice. As has been previously reported,^18^ GPs felt that the provision of guidance in long form could be initially off-putting, so ways of delivering this in smaller chunks or alternative formats would be useful. Of note, a digital format is currently in production. Some GPs practising in areas of high social disadvantage felt that further adaptation would be needed for more disadvantaged populations, citing, for example, that the advice of letting children choose between two healthy options may not be affordable to everyone. Nonetheless, in general, they considered that the guidance could very easily be integrated into their standard care, and that it fitted well with a more compassionate frame for discussing childhood weight and obesity that they are trying to establish. This collaborative approach and positive language use throughout the guidance is supported by previous work.^8^ Patients have long reported perceiving weight stigma from health professionals in healthcare settings.^28^ Providing written information to patients to enhance understanding following consultations about sensitive health topics have been useful in other settings^29^ and this study suggests that GPs at least see the present resource as an opportunity for communicating that they are taking a non-stigmatising approach to weight discussions.

Consistent with recent work in other countries,^30^ the GPs in this study strongly believed that managing childhood obesity was within their role, but requires an integrated multidisciplinary approach. As such, they felt that any message to parents should be consistent across primary, secondary, and community care, and thus that guidance such as this should be introduced simultaneously across multidisciplinary teams to ensure parents get a consistent message. Implementation of guidance is challenging both within and across professions,^31^ and our past research shows that even within very specific policy implementation,^15,32^ the awareness and use of guidance can be limited if not knitted into standard practice.

### Implications for research and practice

The findings indicate an appetite among GPs for integrating the guidance into routine primary care. Further research is needed to evaluate its effect on children’s health and wellbeing, as well as its practical implementation. Relevant child outcomes include whether parental conversations are prompted and the effects of these conversations on wellbeing. For example, they may support children who have experienced teasing or bullying, although it may also risk causing distress. The guidance may additionally influence parental actions around health behaviours, such as family dietary changes or increased physical activity, with or without involving the child directly.

From a practice perspective, future research should examine whether guidance use increases the likelihood of GPs raising weight issues, its effect on consultation time, influence on doctor–patient relationships, and potential need for follow-up appointments. Additionally, exploration of different delivery formats, including digital tools, is warranted to optimise usability and effectiveness.

## Data

The dataset relied on in this article is available from the corresponding author on reasonable request.
